# *phoD*-harboring bacterial community composition dominates organic P mineralization under long-term P fertilization in acid purple soil

**DOI:** 10.3389/fmicb.2022.1045919

**Published:** 2022-11-24

**Authors:** Ming Lang, Haoming Li, Prakash Lakshmanan, Yuanxue Chen, Xinping Chen

**Affiliations:** ^1^College of Resources and Environment, Academy of Agricultural Sciences, Southwest University, Chongqing, China; ^2^Interdisciplinary Research Center for Agriculture Green Development in Yangtze River Basin, Southwest University, Chongqing, China; ^3^Sugarcane Research Institute, Guangxi Academy of Agricultural Sciences, Nanning, China; ^4^Queensland Alliance for Agriculture and Food Innovation, The University of Queensland, St Lucia, QLD, Australia; ^5^College of Resource Sciences, Sichuan Agricultural University, Chengdu, China

**Keywords:** long-term P fertilization, organic P speciation, *phoD*-harboring bacteria, co-occurrence network, keystone taxa, alkaline phosphatase

## Abstract

**Introduction:**

A better understanding of the regulatory role of microorganisms on soil phosphorous (P) mobilization is critical for developing sustainable fertilization practices and reducing P resource scarcity. The *phoD* genes regulate soil organic P (Po) mobilization.

**Methods:**

Based on the long-term P application experiments in acid purple soil of maize system in Southwest China (started in 2010), the experiment included five P levels: 0, 16, 33, 49, and 65.5 kg P hm^–2^ (P0, P16, P33, P49, and P65.5, respectively). The molecular speciation of organic P in soil was determined by 31P-nuclear magnetic resonance (NMR), high-throughput sequencing technology, and real-time qPCR were used to analyze the bacterial community and abundance of *phoD*-harboring bacterial genes, exploring the bacterial community and abundance characteristics of *phoD* gene and its relationship with the forms of Po and alkaline phosphatase (ALP) activity in the soil.

**Results:**

The results showed that the orthophosphate monoesters (OM) were the main Po speciation and varied by P fertilization in acid purple soil. ALP activity decreased as P fertilization increased. Co-occurrence network analysis identified the overall network under five P fertilizations. The keystone taxon base on the network showed that *Collimonas*, *Roseateles*, *Mesorhizobium*, and *Cellulomonas* positively correlated with both OM and Po. The random forest showed that *Cellulomonas*, *Roseateles*, and *Rhodoplanes* were the key predictors for ALP activity. The keystone taxon was a more important predictor than the dominant taxon for ALP, OM, and Po. The structural equation model (SEM) showed that soil organic matter (SOM), available P (AP), and OM were the main factors influencing the ALP by reshaping *phoD*-harboring bacteria alpha diversity, community composition, and *phoD* abundance.

**Discussion:**

The *phoD*-harboring bacterial community composition especially the keystone taxon rather than alpha diversity and abundance dominated the ALP activity, which could promote P utilization over an intensive agroecosystem. These findings improve the understanding of how long-term gradient fertilization influences the community composition and function of P-solubilizing microorganisms in acid purple soil.

## Introduction

Excessive mineral P input in agricultural soil beyond the crop’s need to boost productivity is widespread in intensive agroecosystems (MacDonald et al., 2011; Lou et al., 2015; Zhu et al., 2018). P fertilizer is applied in the field soil, the majority of which is fixed in soils rather than absorbed by plants (Kochian, 2012). The residual P caused by the continuous accumulation resulted in the potential eutrophication of surface water (Powers et al., 2016). It is estimated that the global mineral P reserves are evaluated to be completely depleted by 2100 (Penuelas et al., 2013). Therefore, the ecologically friendly management of fertilization for reducing the use of P and improving P use efficiency is urgently needed for both sustainable agricultural production and environmental health.

Organic P is an important part of the soil P pool. The average content of total P in the topsoil of agroecosystems globally is estimated to be 1,762 kg ha^–1^, of which the Po accounts for 42% (747 kg ha^–1^) (Stutter et al., 2012; Menezes-Blackburn et al., 2018). The orthophosphate monoesters (OM) content measured by ^31^P-nuclear magnetic resonance (NMR) showed no consistent results (unchanged, increased, and decreased) with applied long-term Pi fertilization (Gatiboni et al., 2013; Cade-Menun et al., 2017; van der Bom et al., 2019). Further, little is known about the effect of long-term mineral P fertilization on the Po speciation in acid purple soil commonly seen in Southwest China.

Microorganisms play a key role in the biogeochemical degradation of Po, as they can excrete extracellular enzymes such as phosphatase, including alkaline phosphatase (ALP) and acid phosphatase, in soils. Three bacterial homologous genes encoding phosphatase involved in the production of ALP (*phoA*, *phoD*, and *phoX*) have been identified (Kathuria and Martiny, 2011). *phoD* is the most common ALP gene, which is considered the key ALP gene in soil (Tan et al., 2013), and P supply level is an important factor influencing the community composition of *phoD*-harboring bacteria. With sufficient soil P, *Actinobacteria* and *Cyanobacteria* are the most important groups that mediate Po mineralization in rice soil, while *Methylbacillus* species mainly facilitate Po mineralization under the condition of sufficient P (Wei et al., 2019). P supply level and carbon source provided by roots synergistically affect the community composition of *phoD*-harboring bacteria in the rice rhizosphere. Among them, *Nostocales* dominate under low P conditions, while *Rhizobiales* and *Rhodospirillales* dominate under moderate and high P conditions, respectively. These dominant populations mainly use the carbon source provided by plants (Long et al., 2018), which suggests that the soil P supply level and plants co-determine the community composition of *phoD*-harboring bacteria. It was found that *phoD*-harboring bacteria promote ALP secretion and thus mineralization of Po in both the traditional intensive agricultural and natural grassland soils (Fraser et al., 2015). Further research found that ALP secreted by microorganisms mainly mineralizes unstable forms of Po (Luo et al., 2017), which showed that the community characteristics of *phoD*-harboring bacteria are closely related to Po speciation. Therefore, under different P supply levels, the community characteristics of *phoD*-harboring bacteria (including alpha diversity, beta diversity, community composition, network characteristics, and keystone taxon), Po speciation, ALP activity, and their interactions need more detailed and systematic study.

This study aimed to provide a systematic knowledge of how the microbiota relevant to Po mineralization was affected by gradient P fertilization in acid purple soil. We hypothesized that moderate P fertilizer application would increase the *phoD*-harboring bacterial α-diversity and promote the complexity and stability of microbial network structure with conditions favorable for Po mineralization in intensive maize crop production. To test this hypothesis, we studied the *phoD*-harboring bacterial abundance, α-diversity, microbial community composition, structure, network, and keystone taxa in the soil as affected by different rates of P fertilizer applied to maize crops. For this purpose, soil samples were collected from a long-term field experiment that had been continuously fertilized for 10 years, and the *phoD* gene was used to quantify and identify the soil bacterial abundance, communities, and keystone taxon.

## Materials and methods

### Field site situation and experiment design

The experimental site is located at the Yaan Experimental Station of the Sichuan Agricultural University, Yaan city, Sichuan Province, China (29°58′59.1″N, 102°58′56.4″E). Yaan city is a typical area of intensive agriculture on acid purple soil in Southwest China, and more than 80% of the agricultural fields in the county follow a wheat-maize rotation. The site is located at an altitude of 600 m, and it has a subtropical monsoon humid climate with annual average precipitation and temperature of 1,732 mm and 13.2°C, respectively. It receives on average 218 days of rainfall annually, with about 50% of precipitation falling in summer and about 30% during the wheat growing season. The soil is a purple moist rudiment soil, and its basic soil properties are shown in Supplementary Table 1.

### Experimental design

The field experiment was conducted in October 2010 in a farmland under monoculture maize cropping for 10 years without fertilization just prior to this experiment. During the entire duration of this field experiment, in each rotation year, maize (variety Zhongyu3) was planted at the beginning of April and harvested at the end of August. To build up a range of soil AP levels quickly in the experimental area, 0, 16, 33, 49, and 65.5 kg P ha^–1^ were applied for maize. Each treatment had three replicated plots, with each plot having an area of 26.6 m^2^ (2.8 m × 9.5 m).

At the end of each crop season, straw was removed before the next maize was sown, fertilizers were then broadcasted and mixed into the topsoil by plowing: P fertilizer was applied as calcium superphosphate, N as urea (75 kg N ha^–1^), and K as potassium sulfate (50 kg K ha^–1^). About 150 kg N ha^–1^ as urea was top-dressed at the jointing stage for wheat and at the 12-leaf stage for maize. To compensate for the low precipitation in the growing season, maize was flood irrigated with 50 mm of water once at sowing.

### Sampling and analysis

Soil samples were taken on the maize silking stage on 2 July 2019. From each plot, soil cores from five different locations were taken from 20 cm depth between plant rows, which were combined as a single replicate sample. The combined soil sample was sieved with 2 mm mesh and then divided into two portions. One portion was stored at –20°C for DNA extraction and subsequent amplicon sequencing, and the other portion was air-dried for the analysis of physiochemical properties. There were a total of 15 soil samples, and each soil sample was analyzed as two parallel samples. Soil available P (AP) was extracted with 0.5 M NaHCO3 at pH 8.5 and then determined by colorimetry (Olsen and Sommers, 1982). The acid digestion method was used to determine the total P in soil (Tiessen and Moir, 1993). Po was determined by the ignition method (Saunders and Williams, 1955). Pi was calculated as the total P minus Po. Soil pH was determined in a 1:2.5 (soil/water ratio) suspension with a pH meter. Soil organic carbon (SOC) was determined by the external-heat potassium dichromate oxidation-colorimetric method (Nelson and Sommers, 1983). Soil total N (TN) was measured by dry combustion with an elemental analyzer (Vario max CN, Elementar, Hanau, Germany). The ALP was determined using the method described by Tabatabai (1994).

### Soil P speciation by solution-state ^31^P-NMR spectroscopy

P speciation in soils was identified by ^31^P-NMR spectroscopy following the method of Cade-Menun and Preston (1996). The MestReC software (version 4.9.9.9) was used to correct and process the ^31^P spectra. The free induction decays for solution-state ^31^P-NMR spectra were transformed by using a four-fold zero filling and a line broadening of 6 Hz. The individual peaks of the signal areas were calculated from a deconvolution process. The MDP was used as the internal standard in the solution for NMR analyses and it also calibrated the frequency axis, standardized data, and performed a quantitative assessment of P speciation. Signals were assigned based on the references (Cade-Menun, 2005; McLaren et al., 2015).

### DNA extraction and gene quantification

DNA extract was used as 0.25 g soil per sample with a DNeasy PowerSoil (QIAGEN, Germany) according to the manufacturer’s instructions. A spectrophotometer (Nanodrop ND-1000, Thermo Scientific, Waltham, MA, United States) was used to analyze the quality of extracted DNA. QuantiFluor^®^ dsDNA system (Promega, Madison, WI, United States) was used to determine the concentration of DNA based on fluorometric analysis with a microplate reader (Spectramax M5, Molecular Devices, Sunnyvale, CA, United States). DNA samples were stored at −20°C until they were used for analyses. Real-time quantitative PCR (qPCR) was used to determine the bacterial *phoD* gene abundance with the primers of ALPS-F730/ALPS-R1101 (CAGTGGGACGACCACGAGGT/GAGGCCGATCGGCATG TCG) (Sakurai et al., 2008). The real-time qPCR system was in a 20-μl reaction system with triplicates. LightCycler 480 (Roche, Basel, Switzerland) was used to quantify the gene copy number. The PCR reaction conditions used for the *phoD* gene are as follows: 3 min at 94°C, 40 cycles at 95°C for 15 s, 56°C for 30 s, 72°C for 15 s, and finally 1 min at 72°C. The standard curve was prepared in triplicate with six serial 10-fold dilutions of standard plasmids carrying the *phoD* gene, and the gene copy number was calculated based on the concentration of plasmid DNA and the base pairs therein (vector plus primer). The efficiency of each reaction was 92.41%.

### PCR amplification, high-throughput sequencing, and data analysis

The primers ALPS-F730/ALPS-R1101 were used to amplify the bacterial *phoD* gene (Sakurai et al., 2008), which was the same as in the real-time qPCR experiment. The protocol of the amplification for the bacterial *phoD* gene was as follows: 3 min at 94°C, followed by 40 cycles at 95°C for 15 s, 30 s at 56°C, 72°C for 15 s, and finally 1 min at 72°C. Universal DNA Purification Kit (Tiangen, Beijing, China) and the QuantiFluor^®^ dsDNA system were used to purify and quantify the PCR products, respectively. The purified amplicons were pooled in equimolar volumes and subjected to paired-end sequencing using the Illumina Hiseq 2500 platform (Novogene, Beijing, China).

All the raw sequencing data were purified by the Quantitative Insights Into Microbial Ecology (QIIME) system. The raw data filtered the reads which contained ambiguous-based nucleotide mismatches within the primer or barcode, and reads shorter than 200 bp would be removed before analysis. UCHIME was used to identify and eliminate chimeric sequences. UCLUST clustering was used to cluster Operational taxonomic units (OTUs) at a cutoff of 75% similarity (Fraser et al., 2015). The RDP classifier was used to retrieve and classify the representative sequences. Singletons were removed during this clustering process. Subsequently, Local Blast 2.2.27+^1^ was used to taxonomically assign the representative OTUs, and the P-solubilizing bacterial taxon was annotated with critical criteria (E < 1 × 10^–10^ and sequence identity >99%) (Zheng et al., 2017). In our study, a total of 3,103,247 raw sequences were obtained for high-quality filtered sequences. All sequences were deposited in the NCBI Sequence Read Archive database (accession number PRJNA787763).

### Calculations and statistical analysis

One-way analysis of variance was used to examine the significant differences in soil physiochemical properties, abundance, and α-diversity of *phoD*-harboring bacterial data between the five gradient fertilization treatments and Duncan’s multiple range test at the 0.05 level were used to compare with the mean values with SPSS version 20.0 software package (SPSS, Inc., Chicago, IL, United States). Permutational multivariate analysis of variance was used to investigate the *phoD*-harboring bacterial community affected by gradient P fertilization (Anderson, 2001), which was performed by a Vegan package in R (version 3.3.1). The effects of gradient fertilization on *phoD*-harboring bacterial communities were determined by the analysis of similarities. The “analysis of similarities” function statistically tests the significant effect among treatments. The effects of the soil physiochemical on *phoD*-harboring bacteria were determined by Redundancy analysis (RDA) using Vegan; the RDA model selected the significant environmental variables (*P* < 0.05) (measured based on 999 permutations). Spearman correlation coefficient analyses were conducted to conclude the networks with *phoD*-harboring bacterial OTU-OTU interaction as described by Faust et al. (2012) and Pérez-Jaramillo et al. (2019). The OTUs were selected from the top 500 at the taxonomic level to perform the network analysis. The classification of community composition level was genus. Co-occurrence network visualization was achieved with the Gephi platform (version 0.9.2) (Bastian et al., 2009). The importance of Po, OM, and ALP to keystone taxon was estimated by the Random forest model (Trivedi et al., 2016). Mean squared error (MES%) was used to select the significant important variables (Breiman, 2001). A3 package was used to evaluate the *R*^2^ values for cross-validation and the significance of the model. The “rfPermute” package was used to assess the significance of variables. The structural equation modeling (SEM) framework was conducted to estimate the relationships among soil physiochemical properties (including P rates, SOM, AP, and OM), *phoD* gene abundance, alpha diversity, community composition of the *phoD*-harboring bacteria, and ALP activities using AMOS software (IBM SPSS AMOS 17.0.0). The maximum likelihood estimation and the chi-square test (v2) were used to conduct the best-fit model, the overall goodness of the model fit used the root mean square errors of approximation (RMSEA < 0.05) and comparative fit index (CFI) (>0.90).

## Results

### Soil properties and P status

**FIGURE 1:**
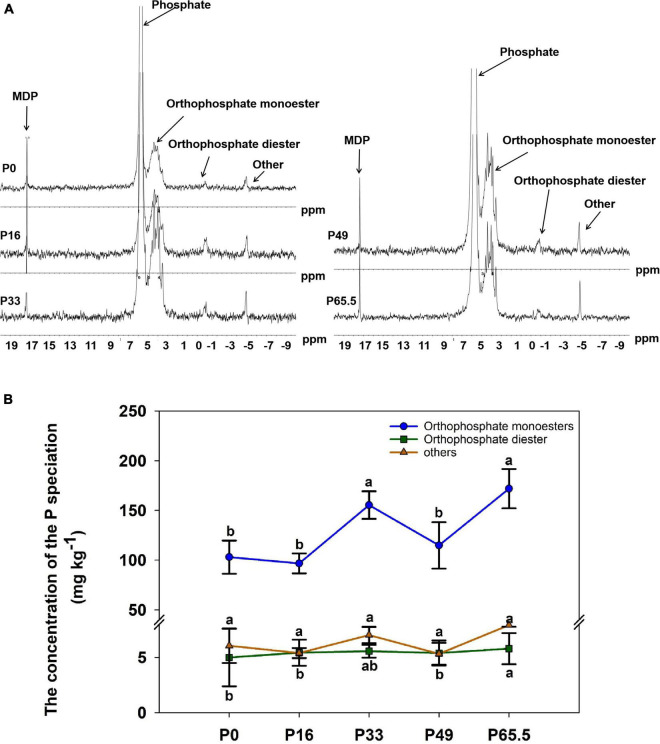
The concentration of the P speciation under gradient P fertilization rates. **(A)** Solution ^31^P NMR (nuclear magnetic resonance) spectra on NaOH-EDTA extracts for the five P fertilization rates. Nuclear magnetic resonance can be used to identify the exact molecular forms of P in soils. These include phosphate (δ 5.83 ppm), orthophosphate monoester (OM) (δ 4.75 ppm), orthophosphate diester (OD) (δ –0.44 ppm), and others (δ –4.76 ppm). **(B)** Line charts based on ^31^P NMR. The data are based on the concentration of organophosphorus molecular form at five P fertilization rates. P0, P16, P33, P49, and P65.5 represent 0, 16, 33, 49, and 65.5 kg P ha^–1^ applied, respectively.

P fertilization had significant effects on soil properties (Table 1). Soil pH decreased significantly as the P fertilization rate increased; SOM, TN, AK, AP, Pi, Po, and Pt were increased as the P fertilization rate increased (Table 1). ^31^P-NMR spectra for the soil samples are shown in Figure 1A. Po speciation measured by ^31^P-NMR showed that the OM was the main organic species and it increased with increased P fertilization. A similar pattern was evident for orthophosphate diester (OD) as well (Figure 1B).

**TABLE 1:** Physicochemical properties of acidic purple soil under gradient P fertilization rate.

Physicochemical properties	P0	P16	P33	P49	P65.5
pH	6.50 ± 0.03 a	6.62 ± 0.04 a	6.02 ± 0.1 b	5.74 ± 0.01 c	5.81 ± 0.03 c
SOM (g kg^–1^)	29.9 ± 0.3 d	31.3 ± 0.5 c	35.5 ± 0.8 b	37.7 ± 0.2 a	38.4 ± 0.3 a
AP (mg kg^–1^)	17.1 ± 1.3 d	26.3 ± 1.8 c	60.2 ± 4.4 b	73.8 ± 1.7 a	72.3 ± 3.0 a
TN (g kg^–1^)	1.71 ± 0.02 e	1.84 ± 0.03 d	2.06 ± 0.03 c	2.12 ± 0.02 b	2.23 ± 0.01 a
AK (mg kg^–1^)	157.0 ± 5.8 c	163.0 ± 2.9 bc	174.0 ± 5.1 ab	181.0 ± 5.7 a	178.0 ± 2.6 a
Pi (mg kg^–1^)	56.6 ± 2.1 d	68.6 ± 2.6 c	129.8 ± 6.5 b	154.3 ± 0.8 a	157.2 ± 3.6 a
Po (mg kg^–1^)	23.0 ± 1.1 d	32.1 ± 4.0 c	75.2 ± 1.0 a	69.0 ± 4.8 a	70.0 ± 1.8 a
Pt (mg kg^–1^)	79.6 ± 1.0 d	100.6 ± 6.5 c	205.0 ± 7.2 b	223.3 ± 5.1 a	227.1 ± 1.9 a

Values are means ± SD of six replicates. Different lowercase letters in the same column indicate significant differences between samples (*P* < 0.05). P0, P16, P33, P49, and P65.5 represent 0, 16, 33, 49, and 65.5 kg P ha^–1^, respectively. SOM, soil organic matter; AP, available phosphorus; TN, total nitrogen; AK, available potassium; Pi, inorganic phosphorus; Po, organic phosphorus; Pt, total phosphorus.

### Abundance of *phoD*, community diversity, and composition of *phoD*-harboring bacterial communities under long-term gradient P fertilization

**FIGURE 2:**
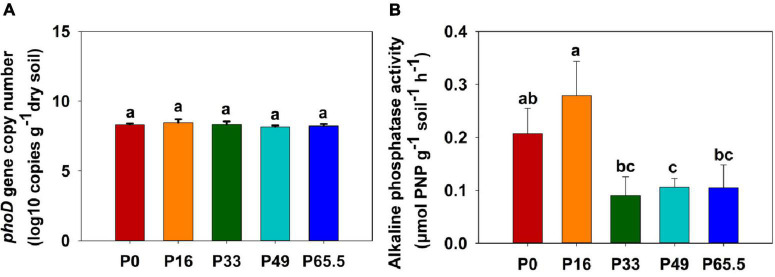
*phoD* gene abundance **(A)** and alkaline phosphatase (ALP) **(B)** in soils treated with five phosphorus fertilization rates. Small letters indicate a significant difference (*P* < 0.05 after the Duncan test) between P levels. P0, P16, P33, P49, and P65.5 represent 0, 16, 33, 49, and 65.5 kg P ha^–1^, respectively.

**FIGURE 3:**
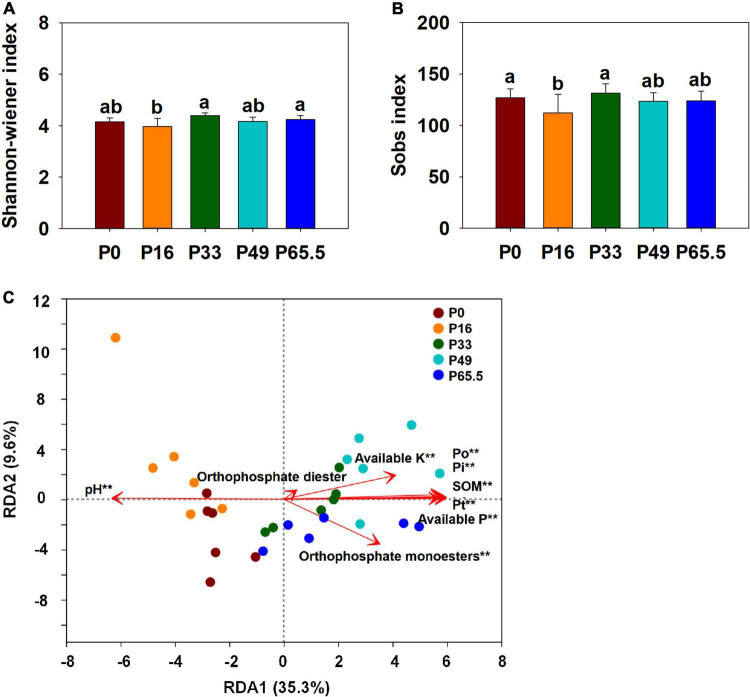
Alpha diversity **(A,B)** and redundancy analysis (RDA) **(C)** of *phoD*-harboring bacteria under gradient P fertilization. In **(A,B)**, small letters indicate significant differences (*P* < 0.05 after the Duncan test) between P levels. In **(C)**, RDA was used to explore the relationship between the entire *phoD*-harboring bacterial community and selected soil properties. **Correlation is significant at the 0.01 level. P0, P16, P33, P49, and P65.5 represent 0, 16, 33, 49, and 65.5 kg P ha^–1^, respectively.

**FIGURE 4:**
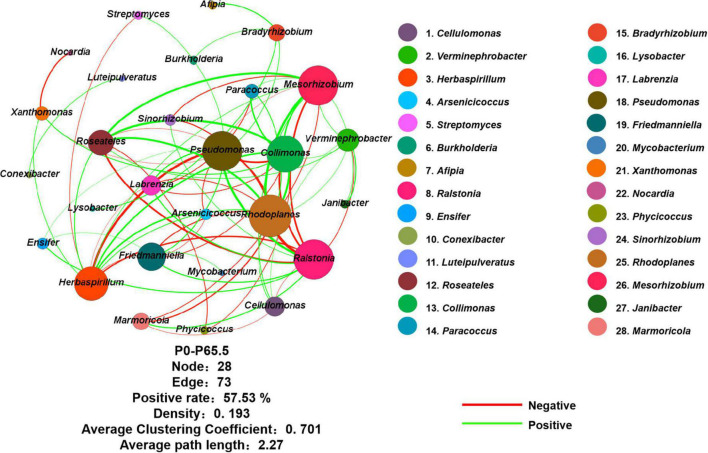
Network of *phoD*-harboring bacteria based on Spearman correlation analysis from OTU profiles. The network consists of five gradient phosphorus fertilization rates (including P0, P16, P33, P49, and P65.5). The color of the nodes represents the taxa of the genus classification. Red lines represent a positive correlation, and green lines indicate a negative correlation. Node, edge, positive rate, density, average clustering coefficient, and average path length of the topological parameters were shown below the network. P0, P16, P33, P49, and P65.5 represent 0, 16, 33, 49, and 65.5 kg P ha^–1^, respectively.

The *phoD* gene abundance was not significantly altered by the gradient P fertilization (Figure 2A), whereas the activity of ALP was decreased as P fertilization decreased (Figure 2B). The alpha diversity was estimated by the Sob index and the Shannon–Wiener index. Generally, there were no significant differences in the *phoD*-harboring Sob index and the Shannon–Wiener index among all five treatments except for the P16 treatment (Figures 3A,B), but the *phoD*-harboring bacterial community structure was significantly affected by P fertilization (Figure 3C and Supplementary Table 3). Based on the composition of all soil samples, the dominant *phoD*-harboring bacterial phyla were Proteobacteria and Actinobacteria, reaching up to 84.7 and 14.7%, respectively (Supplementary Figure 1A). The dominant *phoD*-harboring bacterial class was Alpha-proteobacteria, Beta-proteobacteria, and Actinobacteria, reaching up to 59.5, 19.8, and 14.6%, respectively (Supplementary Figure 1B). A total of 10 genera were aligned, and the most dominant genera belonged to *Bradyrhizobium* (43.2%), *Streptomyces* (7.8%), and *Burkholderia* (5.5%) (Supplementary Figure 2). The relative abundance of *Bradyrhizobium* was highest in P0 treatment and it decreased with increasing P fertilization (Supplementary Figure 2A). The relative abundance of Streptomyces increased first and then decreased (Supplementary Figure 2B), but there was no significant difference among five P fertilization treatments (Supplementary Figure 2C). The random forest showed that *Collimonas*, *Rhodoplanes*, *Roseateles*, *Pseudomonas*, and *Mesorhizobium* were the key predictors for OM and Po (Figure 4). Moreover, *Mesorhizobium* and *Burkholderia* were the key predictors of ALP activity (Supplementary Figure 3).

### Network structure and keystone taxa

**FIGURE 5:**
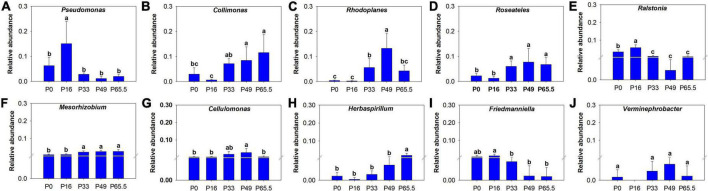
Bar chart of relative abundance of the keystone taxa at genus level under different phosphorus fertilization rates. **(A)** *Pseudomonas*; **(B)** *Collimonas*; **(C)** *Rhodoplanes*; **(D)** *Roseateles*; **(E)** *Ralstonia*; **(F)** *Mesorhizobium*; **(G)** *Cellulomonas*; **(H)** *Herbaspirillum*; **(I)** *Friedmanniella*; **(J)** *Verminephrobacter*. The key species were selected by ranking the top 10 “Degree” values based on the network. P0, P16, P33, P49, and P65.5 represent 0, 16, 33, 49, and 65.5 kg P ha^–1^, respectively.

**FIGURE 6:**
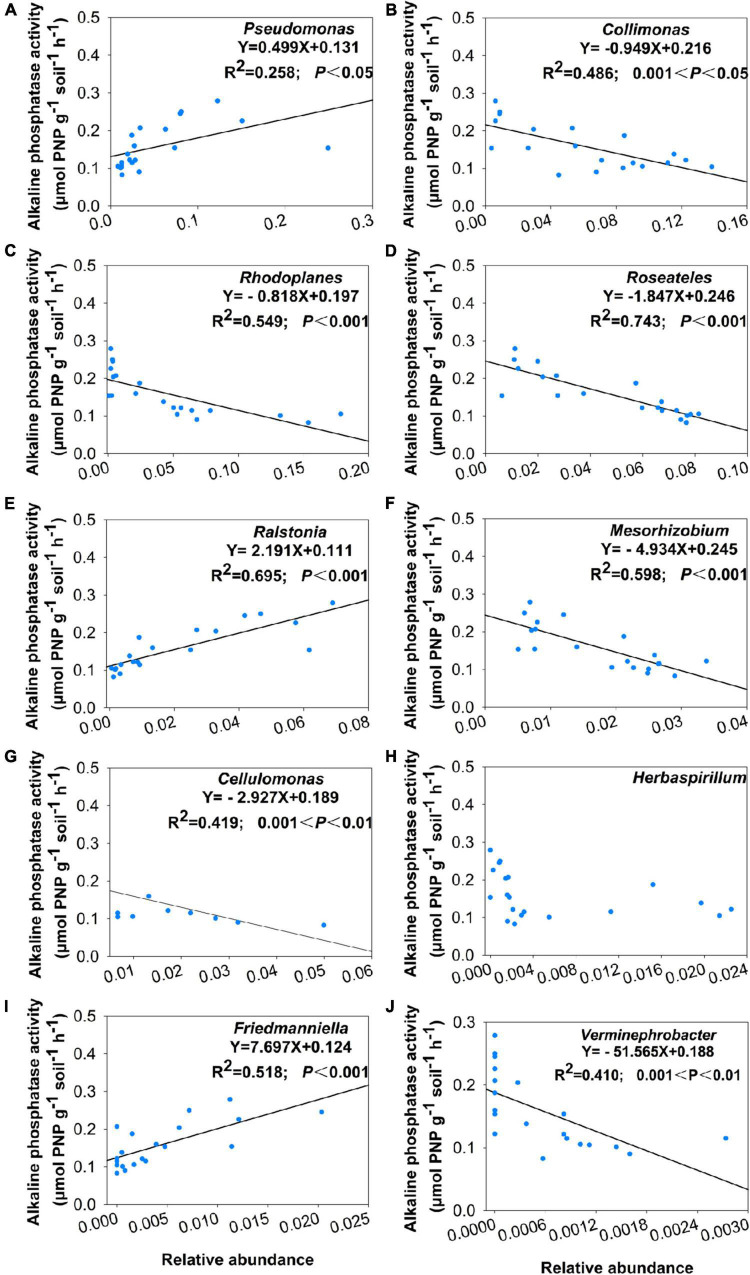
Correlation between alkaline phosphatase activity and relative abundance of keystone taxa which include **(A)** *Pseudomonas*, **(B)** *Collimonas*, **(C)** *Rhodoplanes*, **(D)** *Roseateles*, **(E)** *Ralstonia*, **(F)** *Mesorhizobium*, **(G)** *Cellulomonas*, **(H)** *Herbaspirillum*, **(I)** *Friedmanniella*, and **(J)** *Verminephrobacter*.

**FIGURE 7:**
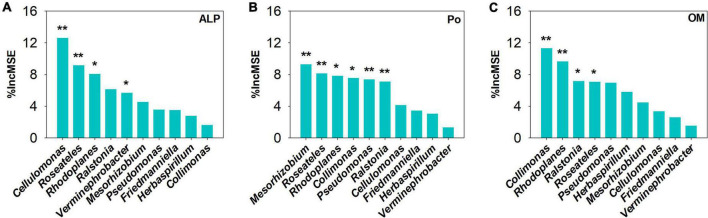
Random Forest analysis to identify the main predictors of keystone taxa on **(A)** alkaline phosphatases (ALP), **(B)** organophosphorus (Po), and **(C)** orthophosphate monoester. The mean decrease in accuracy *(%IncMSE)* was used to indicate the relative importance of each variable for predicting the soil’s total P concentration. *Correlation is significant at the 0.05 level; **correlation is significant at the 0.01 level.

**FIGURE 8:**
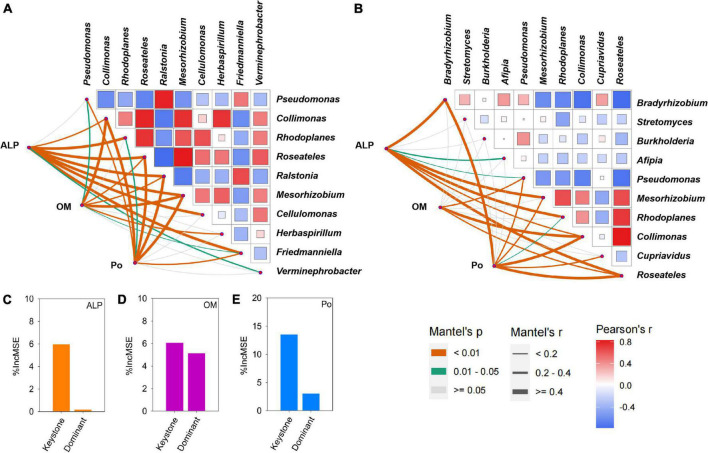
Correlation among alkaline phosphatases (ALP), orthophosphate monoester (OM), and organophosphorus (Po) with the keystone **(A)** and dominant taxon **(B)**. Pairwise comparisons of the keystone and dominant taxon are separately shown in the heatmap with a color gradient to represent Spearman’s correlation coefficients. Statistically significant correlations among ALP, OM, and Po with the keystone and dominant taxon are shown in the corresponding lower left. Edge width corresponds to Mantel’s coefficients. The orange line means *P* < 0.01, the green line means 0.01 < *P* < 0.05, and the gray line means no significant effect. A random forest analysis to identify the importance of keystone taxon and dominant taxon to ALP **(C)**, OM **(D)**, and Po **(E)**.

Using co-occurrence network analysis, we identified the overall network under five P fertilization (Figure 4). The keystone taxon of the overall network was significantly affected by different rates of P fertilization (Figure 5). Among them, *Collimonas*, *Roseateles*, *Mesorhizobium*, and *Cellulomonas* were positively correlated with both OM and Po concentrations (Supplementary Figures 4, 5), while *Pseudomonas*, *Ralstonia*, and *Friedmanniella* were positively correlated with ALP (Figure 6). The random forest analysis showed that *Rhodoplanes* and *Roseateles* were the key predictors for both OM and Po (Figures 7A,B). Moreover, *Cellulomonas*, *Roseateles*, and *Rhodoplanes* were the key predictors of ALP activity (Figure 7C). Distinct network structures were identified separately for five P fertilization treatments used in this study (Supplementary Figure 6). The number of edges and average degree were increased as P fertilization increased (Supplementary Figure 6). The keystone taxon varied with the P fertilization rate (Supplementary Table 3). The majority of the keystone taxa were positively correlated with P forms including AP, Pi, Po, and Pt for P33, P49, and P65.5 treatments, while they were negatively correlated with SOM (Supplementary Figure 7). Both the Mantel test and Random Forest analysis were conducted to identify the importance of keystone and dominant taxon for Po mineralization (Figure 8). Mantel test showed that the ALP, OM, and Po were significantly correlated with the keystone taxon more than that of the dominant taxon (Figure 8A). Random Forest analysis showed that the keystone taxon was the most important predictor than the dominant taxa for ALP, OM, and Po (Figure 8B).

### The main driver of soil properties with *phoD* abundance and community under long-term gradient P fertilization

**FIGURE 9:**
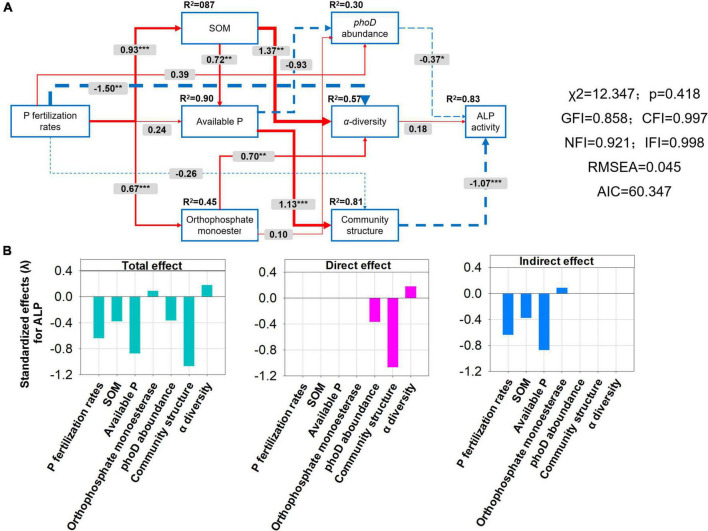
Structural equation model (SEM) **(A)** and bar plots of effect values (total, direct, indirect) on alkaline phosphatases (ALP) activity **(B)** showing the influence of various factors on the correlationship among soil properties [including soil organic matter (SOM), available P (AP), and orthophosphate monoesters (OM)], alpha diversity, *phoD* abundance, community structure, and ALP activity of soil under long-term gradient phosphorus fertilization rates. The width of the arrow indicates the strength of the causal effect. The red and blue arrows indicate the positive and negative relationships between the indicators. The number above the arrow indicates the path coefficient. “*”, “**”, and “*” represent significant path. The percentage above each indicator represents the *R*^2^ value, which is the variance-explained ratio of each variable. The final model fits the data well. The model is: (χ^2^ = 13.343, df = 1.026, CFI = 0.997, IFI = 0.997, RMSEA < 0.043, AIC = 59.343).

A structural equation model (SEM) was constructed to further investigate the relationships among P application rates, *phoD*-harboring bacteria characteristics (including alpha diversity, community composition, and abundance), soil physicochemical factors (including SOM, AP, and OM), and ALP activity (Figure 9). P fertilization rates were significantly and positively related to SOM (path coefficient = 0.93) and OM (path coefficient = 0.67) and were negatively related to alpha diversity (path coefficient = −1.50). SOM and OM were significantly and positively related to alpha diversity, the path coefficients of 1.37 and 0.70, respectively. However, there was no significant correlation between alpha diversity and ALP activity. ALP activity was significantly negatively affected by *phoD* abundance (path coefficient = −0.37). The relationship between community composition (path coefficient = −1.07) and ALP activity was more significant than *phoD* abundance (path coefficient = −0.37) and alpha diversity (path coefficient = 0.18). However, AP was significantly and positively related to the community composition of *phoD*-harboring bacteria (Figure 9A). ALP activity was negatively affected indirectly by P fertilization rates, SOM, and AP, and positively affected by OM. *phoD* abundance and community composition had a direct negative effect on ALP activity. Alpha diversity has a direct positive effect on ALP activity (Figure 8B). In addition, the standardized total effect revealed that the P fertilization rate had a strong negative effect on ALP (Figure 9B).

## Discussion

### Long-term gradient P fertilization affecting P speciation and the alkaline phosphatase activity

In the acidic purple soil, the major limiting factor of maize production is low P availability. Hence, the application of the mineral P fertilization is a common agricultural management practice to improve soil P fertility (Hu et al., 2018). In the current study, P fertilization greatly increased the AP and Pt. The soil mineralogy such as the Fe/Al minerals combine with the pH to co-determine the forms and the availability of P in soil (Hou et al., 2018). Additionally, Po and the main component of Po named OM are generally first increased and then remained unchanged with the increase of P supply level in acid purple soil (Table 1 and Figure 1). The evidence has shown that crop input (root residue) and microbial immobilization are the key ways to form Po in soil (Condron et al., 2014). The changes in root residue and Po show a similar pattern during 10 consecutive years of a wheat-maize rotation system in calcareous soil (Deng et al., 2017; Lang et al., 2021), which may be the case in our present study as well.

The indicator of the potential Po mineralization is ALP activity (Chen, 2003; Gao et al., 2022). ALP in soil hydrolyzes Po and then transforms into the AP forms that plants can absorb (Tabatabai, 1994; Turner and Haygarth, 2005). A previous study shows that P fertilization could enhance the activity of ALP (Fraser et al., 2015). However, the activity of ALP is found suppressed after long-term (30 years) fertilization (Zeng et al., 2022), and our results corroborated this finding, shown in Figure 2B. ALP activity in soil generally origin from microbes, and *phoD*-harboring bacteria secrete ALP, a common enzyme that is widespread in the bacterial kingdom (Ragot et al., 2015). Previous studies indicate that ALP activity is affected by some factors including soil physicochemical properties, the interactions of the soil organism, and the inputs of the nutrient (Bünemann et al., 2011), and the effects of the *phoD*-harboring bacterial community vary for P fertilizer application among sites in specific environments (Fraser et al., 2015; Ikoyi et al., 2018; Chen et al., 2019).

### Long-term gradient P fertilization affecting the abundance and community composition of *phoD*-harboring bacteria

Our results showed that P fertilization significantly affected the *phoD*-harboring bacterial community structure and composition, but not the *phoD* gene abundance. Previous studies demonstrate that the *phoD*-harboring bacterial community is more important than the gene abundance in regulating the expression of phosphatase (Bi et al., 2020; Wan et al., 2020). Meanwhile, in the agroecosystem, Fraser et al. (2015) show that inorganic (mineral) and organic (manure) P fertilizer inputs solely affect the community of *phoD* bacteria but not the copy numbers of *phoD* gene, demonstrating that the community of *phoD*-harboring bacteria is more sensitive than that of gene abundance. The dominant *phoD*-harboring bacteria in our study were Proteobacteria and Actinobacteria, which were in agreement with the previous studies (Lagos et al., 2016; Zheng et al., 2017; Hu et al., 2018). The *Proteobacteria* and *Bacilli* in the *phoD*-harboring communities belonging to the copiotrophic bacteria enriched in the abundant P supplies (with P33, P49, and P65.5 treatments). *Bradyrhizobium* and *Methylobacterium* belonged to the *Rhizobials* enriched in the P33–65.5 treatment, which are generally dominant in soil where the AP is relatively high (Ragot et al., 2016; Long et al., 2018). The ^18^O-DNA-stable isotope probing coupled with high-throughput sequencing was used to characterize the active *phoD*-harboring bacterial communities, showing that the *Bacillus* is the dominant taxa in mineral fertilized soil (Liu et al., 2021), which is consistent with our results. Soil acidification is a common phenomenon under the long-term application of mineral fertilizer in an intensive agroecosystem (Guo et al., 2010; Yang et al., 2018), which was consistent with our results (Table 1). The previous study shows that pH is a key factor in determining Po mineralizing-related gene abundance, which consequently affected corresponding microbial diversity (Wan et al., 2020). The excessive input of the mineral fertilizers caused soil acidification, and soil P forms followed the P fertilization rate, which may alter microbial diversity and soil enzyme activity (Hartmann et al., 2015; Liu et al., 2019). Especially, the dominant taxa *Bradyrhizobium*, nitrogen-fixing symbiotic bacteria which requires a phosphate transport system (Bardin et al., 1996), increases soil AP according to the production of phosphatase or organic acids (Mander et al., 2012; Sharma et al., 2013; Ikoyi et al., 2018). These results reveal that the *phoD*-harboring bacterial community is more sensitive to P fertilization than the gene abundance.

### The effect of the network and keystone taxa on organic P mineralization under long-term gradient P fertilization

The bacterial taxa interaction in niches and the keystone taxon influencing the corresponding bacterial communities can be analyzed by network analysis (Banerjee et al., 2016; Zheng et al., 2017). The keystone taxa obtained from *phoD*-harboring bacteria varied with P fertilization, and some of them were positively or negatively correlated with Po speciation and ALP (Figures 5, 6). *Mesorhizobium*, one of the plant growth-promoting rhizobacteria (PGPRs) (Chen et al., 2021) could produce plenty of metabolites that can promote plant growth (Baba et al., 2021). Ding et al. (2021) demonstrated that the concentrations of sugars and organic acids are coupled with the keystone taxon, such as *Mesorhizobium* and *Bradyrhizobium*. The genus *Collimonas* secreted enzymes such as phosphatases, peptidases, chitinases, and nucleases and had a specific role in weathering and mycophagy (Mela et al., 2012). The *Collimonas* strains can solubilize Pi and produce gluconic acid from glucose, demonstrating that acidification is one of the key mechanisms conducted by these bacteria for mineral weathering (Uroz et al., 2009). These kinds of taxa may have improved Pi dissolution and Po decomposition. The presence of *Pseudomonas*, *Ralstonia*, and *Friedmanniella* positivity correlated with ALP activity, which demonstrates that both genera may play an important role in P deficiency conditions. The previous study showed that ALP activity is revealed to be closely related to *phoD*-harboring bacteria with less abundance, but not with the diversity and abundance of *phoD*-harboring bacteria in subalpine forest ecosystems (Li et al., 2021). Collectively, these results suggest that some specific keystone taxon remain the key drivers of soil ALP activity. Furthermore, the importance of the keystone and dominant taxon analysis conducted by the mantel test and the Random Forest analysis (Figure 8) revealed that the keystone taxon contributed more than that of the dominant taxon to Po mineralization.

### Long-term gradient P fertilization affecting the relationships between *phoD*-harboring bacterial communities and soil biogeochemical properties

Orthophosphate monoesters was the main specification present in acid purple soil, which directly affected the alpha diversity and *phoD*-abundance (Figures 1, 9). The previous study shows that Po availability shapes the diversity of *phoD*-harboring bacteria in agricultural soil, which highlights the key role that Po plays in the *phoD*-harboring bacterial community structuring (Wei et al., 2021). Furthermore, previous studies demonstrated that environmental factors such as pH, AP, and SOC determine the soil bacterial community in different ecosystems (Zhao et al., 2014; Zhou et al., 2015; Chen et al., 2019). More specifically, SOM and AP are the main factors determining the soil *phoD*-harboring bacterial community structures in the wheat fields (Liu et al., 2020). Furthermore, AP and ALP activity shows a significant correlation with the *phoD*-harboring bacterial community structure, and there is a significant correlation between SOC and the *phoD*-harboring bacteria community structure in Northeast China of maize field (Chen et al., 2019). Similarly, SOM and AP significantly affected the *phoD*-harboring bacterial community in our present study (Figure 9), demonstrating their key roles in the *phoD*-harboring bacterial community in acid purple soil.

## Conclusion

In this study, a 10-year field experiment demonstrated that long-term excessive P fertilization significantly decreases soil ALP activity. The keystone taxon contributed more than that of the dominant taxon to Po mineralization. The keystone taxa *Pseudomonas*, *Ralstonia*, and *Friedmanniella* positively correlated with ALP activity. In the acid purple soil, the *phoD*-harboring bacterial communities were mainly driven by SOM, AP, and Po speciation. P solubilizing bacterial community composition especially the keystone taxon played a key role in regulating phosphatase activity than the alpha diversity and abundance, as well as promoting P utilization in intensive agroecosystems. Thus, our results further proved the potential of soil microorganisms for efficient P fertilization management in an intensive agroecosystem. In summary, our results revealed that applying moderate P fertilization could improve the P solubilizing bacterial community and facilitate P mineralization and utilization, which remarkably contributes toward sustainable agriculture. Future studies will verify these findings in more diverse agroecosystems at large spatial scales.

## Data availability statement

The datasets presented in this study can be found in online repositories. The names of the repository/repositories and accession number(s) can be found in the article/Supplementary material.

## Author contributions

ML and XC conceived the study. HL contributed to the data analysis of bioinformatics. ML and HL contributed to the soil sampling and drafting the article. ML, XC, PL, and YC contributed to the critical review and editing of the manuscript. All authors contributed to the article and approved the submitted version.

## Footnotes

1
https://www.ncbi.nlm.nih.gov/sra/?term=PRJNA787763
