# Supplementary material for: phoD-harboring bacterial community composition dominates organic P mineralization under long-term P fertilization in acid purple soil
Source: Front Microbiol. 2022 Nov 24;13:1045919. doi: 10.3389/fmicb.2022.1045919 (PMC9730253; doi:10.3389/fmicb.2022.1045919)
Supplement: Supplementary file 1 [file Data_Sheet_1.docx]

**Supporting information**

***phoD*-harboring bacterial community composition** **dominates organic P mineralization under long-term P fertilization in** **acid purple soil**

**Ming Lang^1,2,#^, Haoming Li^1,#^, Prakash Lakshmanan^2,4,5^, Yuanxue Chen^3^, Xinping Chen^1,2,^***

^1^College of Resources and Environment, Academy of Agricultural Sciences, Southwest University, Chongqing 400716, China

^2^ Interdisciplinary Research Center for Agriculture Green Development in Yangtze River Basin, Southwest University, Chongqing 400716, China

^3^ College of Resource Sciences, Sichuan Agricultural University, Chengdu 611130, China

^4^Sugarcane Research Institute, Guangxi Academy of Agricultural Sciences, Nanning 530007, China

^5^Queensland Alliance for Agriculture and Food Innovation, University of Queensland, St Lucia 4067, QLD, Australia

**^#^ These authors contributed equally to this paper**

*** Corresponding author**

Dr Xinping Chen

Tel: 0086-023-68251082

E-mail address: [chenxp2017@swu.edu.cn](mailto:chenxp2017@swu.edu.cn)

**Table S1** Results of envfit function showing the relationship of soil parameters with redundancy analysis of *phoD*-harboring bacterial communities

| Factors | RDA1 | RDA2 | R^2^ | *P* values |
| --- | --- | --- | --- | --- |
| SOM | 0.9976 | 0.0693 | 0.6889 | 0.001 |
| AP | 0.9961 | 0.0887 | 0.6904 | 0.001 |
| pH | -0.9994 | -0.034 | 0.8109 | 0.001 |
| AK | 0.9407 | 0.3394 | 0.4019 | 0.004 |
| Pi | 0.998 | 0.0638 | 0.7259 | 0.001 |
| Pt | 0.9985 | 0.0544 | 0.7231 | 0.001 |
| Po | 0.9994 | 0.035 | 0.6226 | 0.001 |
| TN | 0.9986 | 0.0538 | 0.6653 | 0.001 |
| OM | 0.8239 | -0.5667 | 0.3994 | 0.004 |
| OD | 0.6914 | 0.7225 | 0.0081 | 0.912 |

The values showed the first principal component axis of *phoD*-harboring bacterial community. P values are based on 999 permutations. Abbreviations: SOM: soil organic matter; AP: available phosphorus; pH: soil acidity-alkalinity; AK: available potassium; Pi: inorganic phosphorus; Pt: total phosphorus; Po: organic phosphorus; TN: total nitrogen; OM: Orthophosphate monoesters; OD: Orthophosphate diester.

**Table S2** Topology parameters for network analysis under gradient P fertilization rate.

| Treatments | Number of node | Number of edge | Number of Positive interactions | Positive rate | Average degree | Diameter | Average path length | Modularity |
| --- | --- | --- | --- | --- | --- | --- | --- | --- |
| P0 | 25 | 23 | 18 | 78.26% | 1.840 | 4 | 2.000 | 0.667 |
| P16 | 27 | 26 | 16 | 61.54% | 1.926 | 4 | 1.926 | 0.657 |
| P33 | 29 | 35 | 26 | 74.29% | 2.414 | 6 | 2.222 | 0.558 |
| P49 | 27 | 33 | 18 | 54.55% | 2.444 | 4 | 1.617 | 0.630 |
| P65.5 | 26 | 32 | 26 | 81.25% | 2.462 | 6 | 2.608 | 0.624 |

Abbreviations: P0, P16, P33, P49, P65.5 represent 0, 16, 33, 49, 65.5 kg P ha^-1^, respectively.

**Table S3** The co-occurrence network analysis of the keystone taxon under gradient P fertilization rates.

| P rate | Genus | P rate | Genus |
| --- | --- | --- | --- |
| P0 | *Ensifer* | P49 | *Ensifer* |
|  | *Pseudomonas* |  | *Xanthomonas* |
|  | *Phycicoccus* |  | *Cellulomonas* |
|  | *Micromonospora* |  | *Nocardia* |
|  | *Methylobacterium* |  | *Cupriavidus* |
|  | *Jatrophihabitans* |  | *Ralstonia* |
|  | *Intrasporangium* |  | *Janibacter* |
|  | *Burkholderia* |  | *Actinoplanes* |
| P16 | *Pseudomonas* |  | *Burkholderia* |
|  | *Burkholderia* |  | *Collimonas* |
|  | *Roseateles* |  | *Jatrophihabitans* |
|  | *Labrenzia* | P65.5 | *Intrasporangium* |
|  | *Nocardia* |  | *Afipia* |
|  | *Ensifer* |  | *Roseateles* |
|  | *Cellulomonas* |  | *Cellulomonas* |
|  | *Phycicoccus* |  | *Pseudomonas* |
| P33 | *Afipia* |  | *Rhodoplanes* |
|  | *Bradyrhizobium* |  |  |
|  | *Luteipulveratus* |  |  |
|  | *Roseateles* |  |  |
|  | *Burkholderia* |  |  |
|  | *Arsenicicoccus* |  |  |
|  | *Rhodoplanes* |  |  |
|  | *Mesorhizobium* |  |  |
|  | *Jatrophihabitans* |  |  |
|  | *Janibacter* |  |  |
|  | *Rhizobacter* |  |  |
|  | *Methylobacteriu* |  |  |

Note: Keystone taxon are annotated at the genus level. Abbreviations: P0, P16, P33, P49, P65.5 represent 0, 16, 33, 49, 65.5 kg P ha^-1^, respectively.


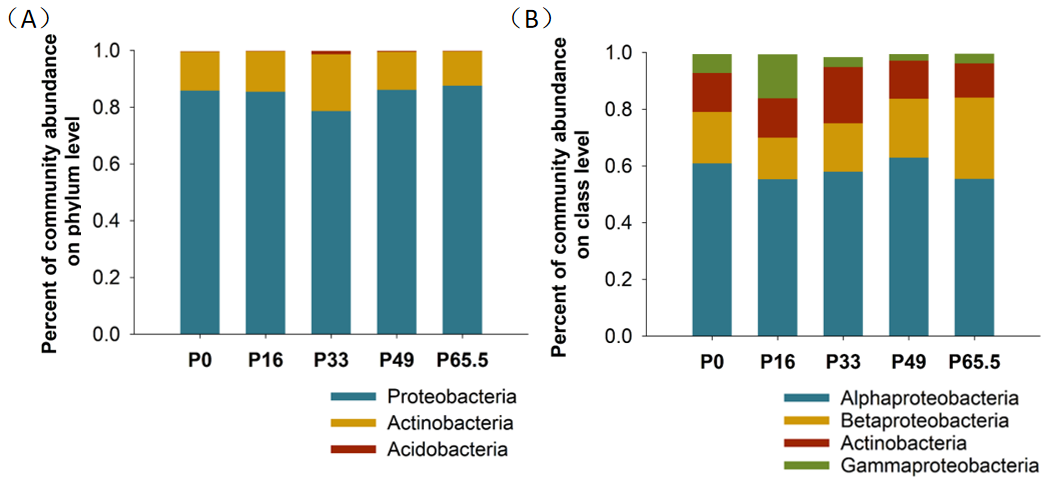


**Figure S1** The community composition of *phoD* bacteria at (A) phylum and (B) class level under gradient phosphorus fertilization rates. Abbreviations: P0, P16, P33, P49, P65.5 represent 0, 16, 33, 49, 65.5 kg P ha^-1^, respective.


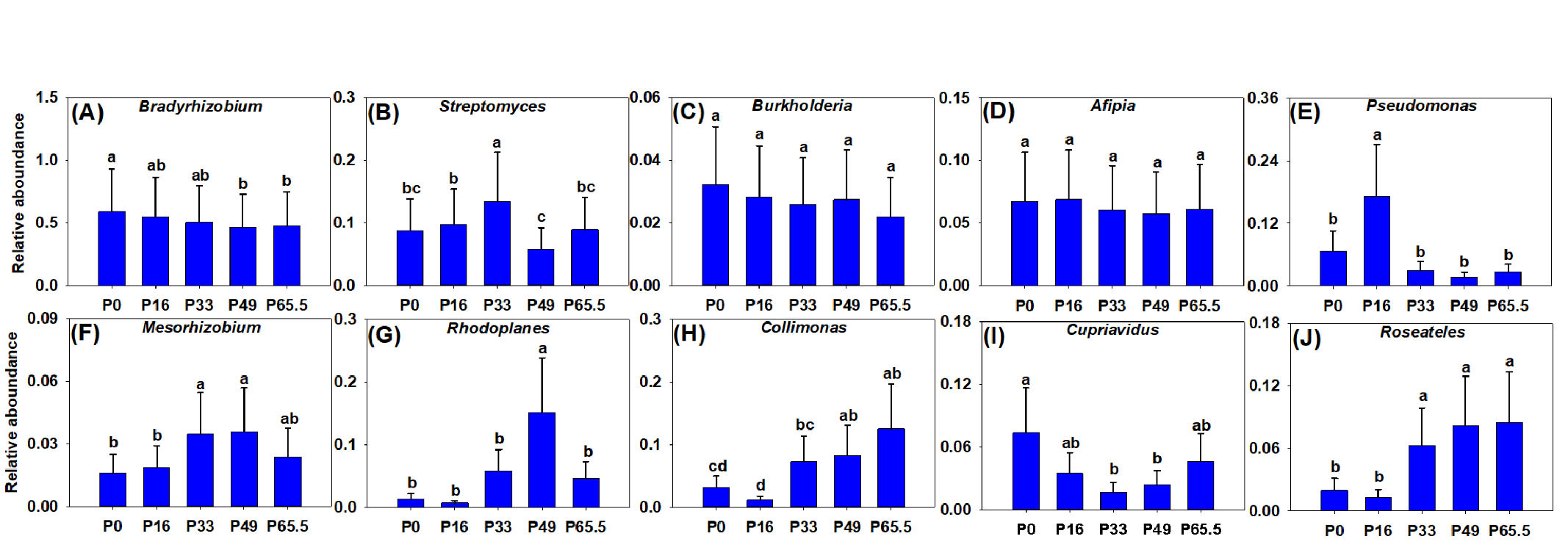


**Figure S2** The relative abundance of *phoD*-harboring bacterial under gradient phosphorus fertilization rates. Different lower case letters denote significantly different on P < 0.05. Abbreviations: P0, P16, P33, P49, P65.5 represent 0, 16, 33, 49, 65.5 kg P ha^-1^, respectively. We screened 10 genera based on the average relative abundance of more than 0.02 under different fertilization levels. These genera are considered the "dominant species", include (A) *Bradyrhizobium*, (B) *Streptomyces*, (C) *Burkholderia*, (D) *Afipia*, (E) *Pseudomonas*, (F) *Mesorhizobium*, (G) *Rhodoplanes*, (H) *Collimonas*, (I) *Cupriavidus*, (J) *Roseateles*.


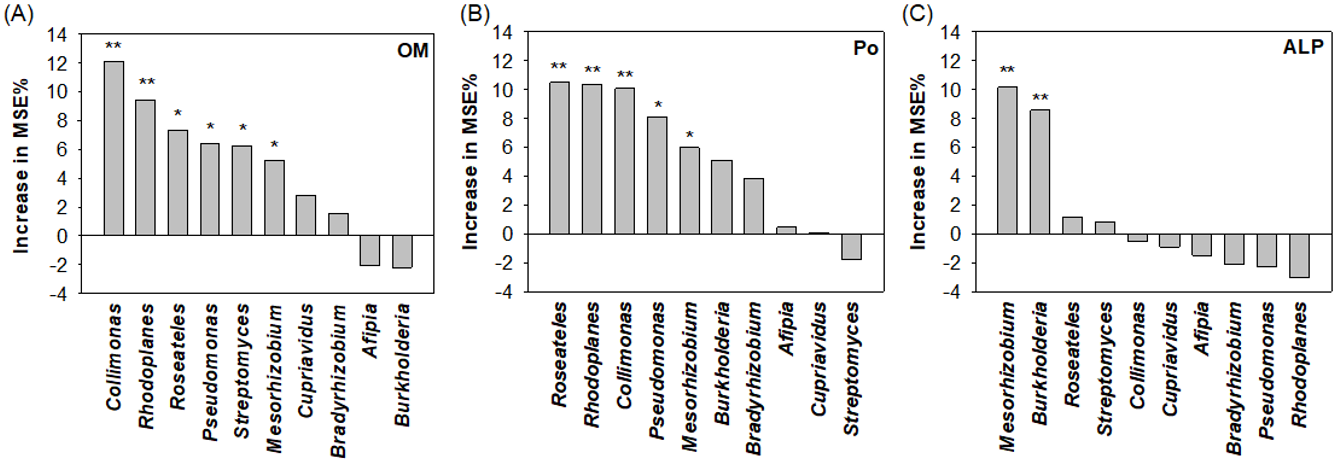


**Figure S3** Predictors of dominant species (genus level) phosphomonoesters (OM), organophosphorus (Po), and alkaline phosphatases (ALP) were analyzed by random forest. Among them, increase in MSE% (Mean Squared error) is an indicator to evaluate the importance of features. The larger the value is, the more important the variable is. "*" means P < 0.05, "**" means P < 0.01.


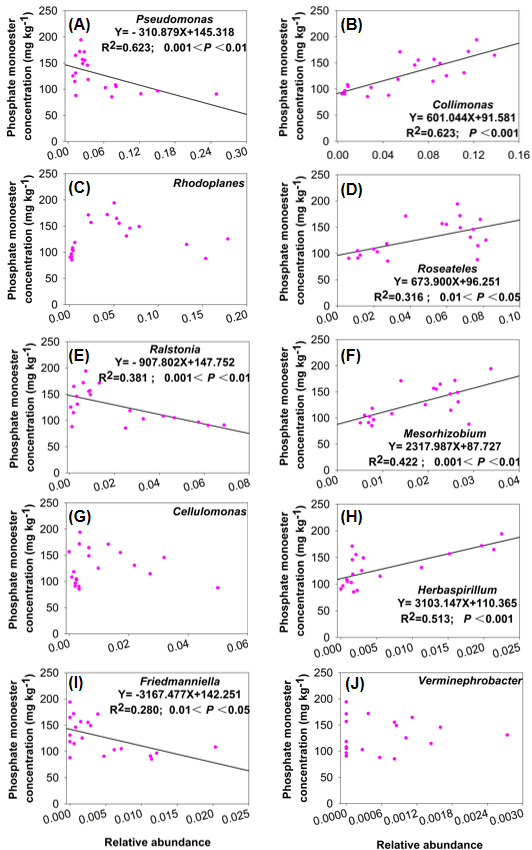


**Figure S4** Correlation between phosphate monoester concentration and relative abundance of keystone taxon including (A) *Pseudomonas*, (B) *Collimonas*, (C) *Rhodoplanes*, (D) *Roseateles*, (E) *Ralstonia*, (F) *Mesorhizobium*, (G) *Cellulomonas*, (H) *Herbaspirillum*, (I) *Friedmanniella*, (J) *Verminephrobacter*. Specifically，OM shows Orthophosphate monoesters.


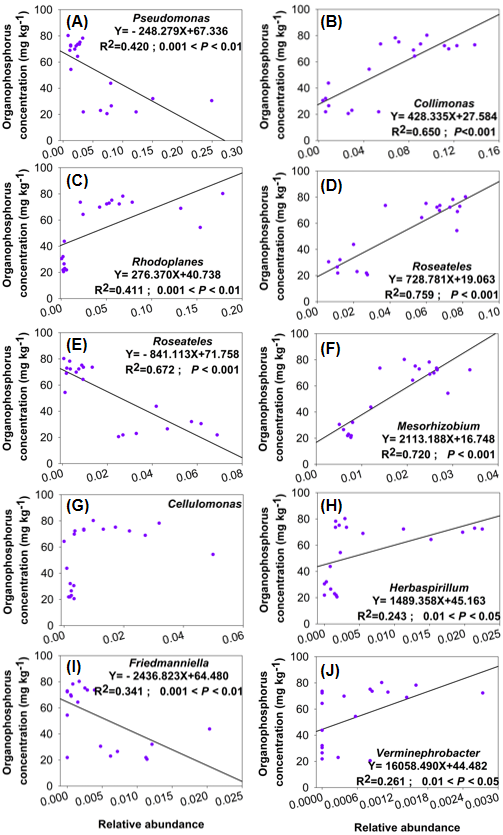


**Figure S5** Correlation between organic phosphorus concentration and relative abundance of keystone taxon including (A) *Pseudomonas*, (B) *Collimonas*, (C) *Rhodoplanes*, (D) *Roseateles*, (E) *Ralstonia*, (F) *Mesorhizobium*, (G) *Cellulomonas*, (H) *Herbaspirillum*, (I) *Friedmanniella*, (J) *Verminephrobacter*. Specifically, Po shows organic phosphorus.


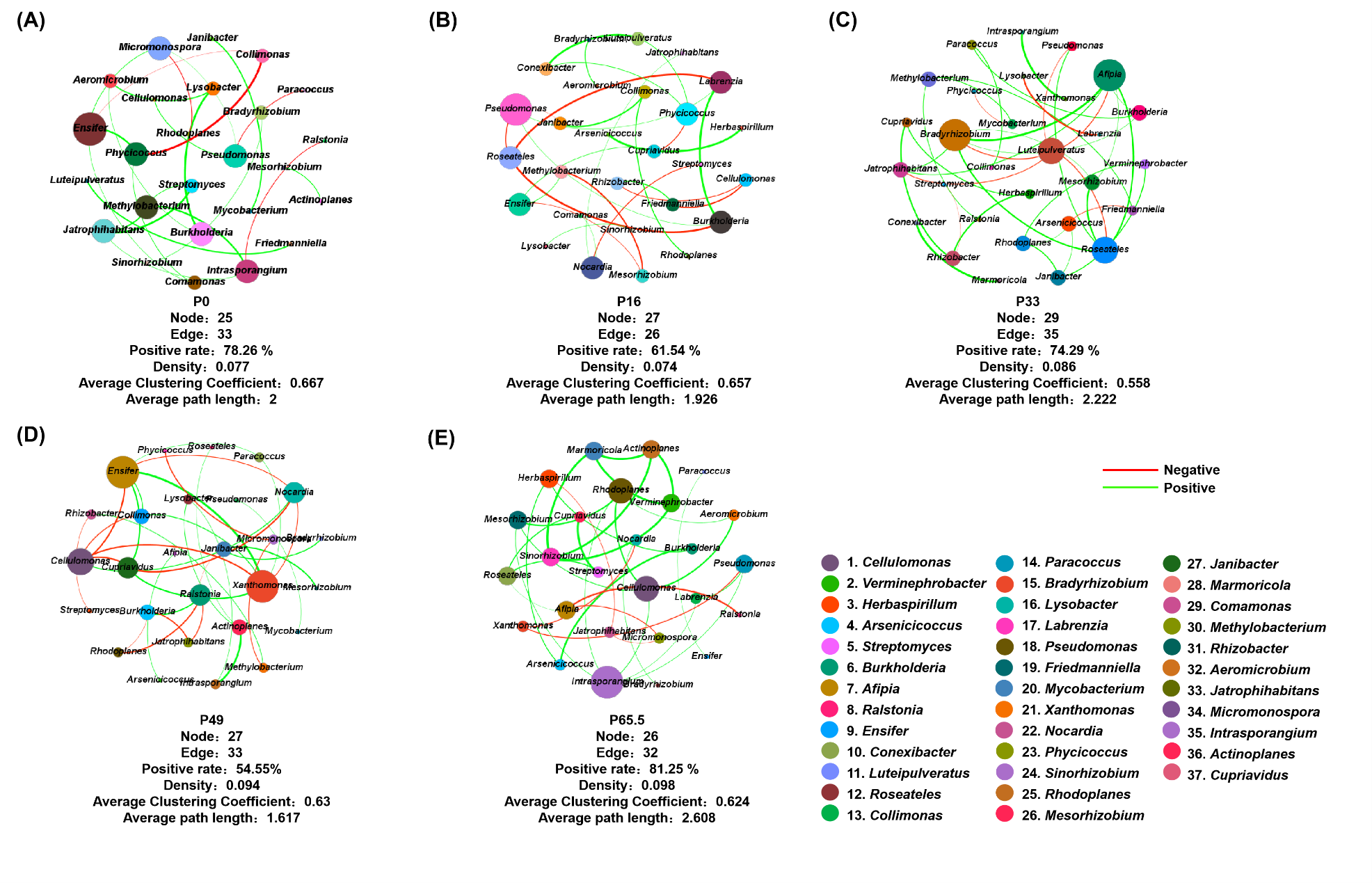


**Figure S6** Network of bacteria depending on P fertilization based on RMT (random matrix theory) analysis from OTU profiles. The size of each node is proportional to the number of connections. The color of nodes represents the taxa on the genus classification. Red lines represent a negative correlation, and green lines indicate a positive correlation. (A) P0; (B) P16; (C) P33; (D) P49; (E) P65.5. Abbreviations: P0, P16, P33, P49, P65.5 represent 0, 16, 33, 49, 65.5 kg P ha^-1^, respectively.


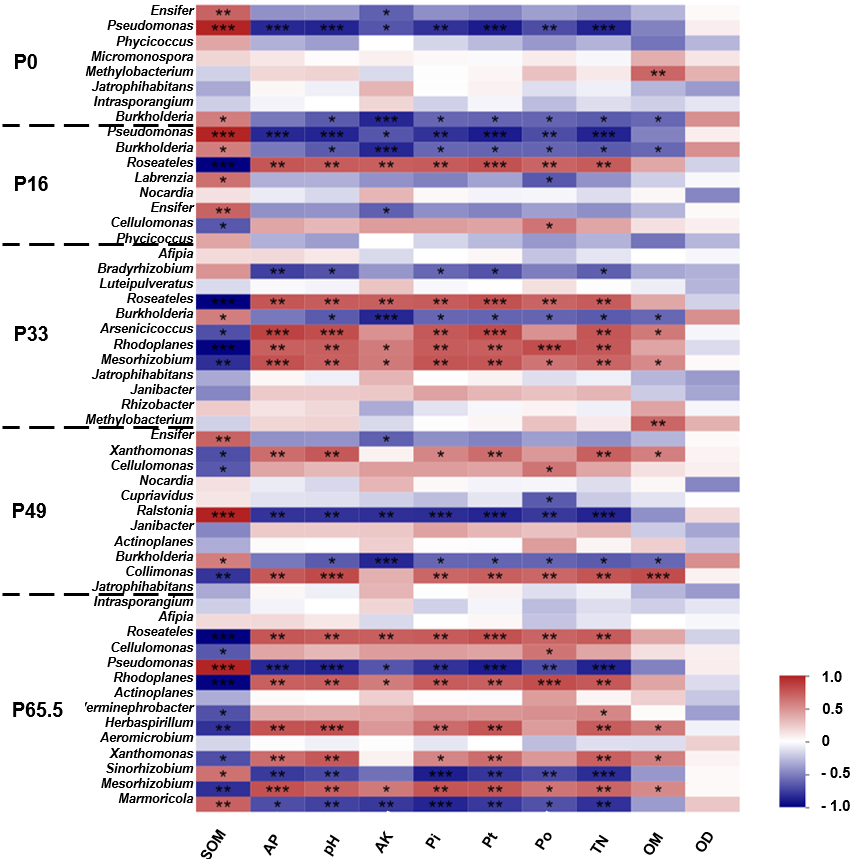


**Figure S7** Heatmaps of correlation coefficients between the soil properties and the keystone taxa. Abbreviations: SOM, soil organic matter. AP, available phosphorus. Po, organic phosphorus. Pi, inorganic phosphorus. Pt, total phosphorus. TN, total nitrogen. “*”indicates a significant correlation P < 0.05. “**” indicates a significant correlation P < 0.01. “***” indicates a significant correlation P < 0.001.
